# Supplementary material for: Ecological status of a freshwater tectonic lake of the indo-burmese province: Implications for livelihood development
Source: PLoS One. 2020 Nov 4;15(11):e0240685. doi: 10.1371/journal.pone.0240685 (PMC7641454; doi:10.1371/journal.pone.0240685)
Supplement: Attachment — Submitted filename: Response to Reviewers and Editors.docx [file pone.0240685.s001.docx]

**In first revision**

1. Please ensure that your manuscript meets PLOS ONE's style requirements, including those for file naming. The PLOS ONE style templates can be found at

<https://journals.plos.org/plosone/s/file?id=wjVg/PLOSOne_formatting_sample_main_body.pdf> and

<https://journals.plos.org/plosone/s/file?id=ba62/PLOSOne_formatting_sample_title_authors_affiliations.pdf>

**We have formatted the manuscript as per the given guidelines.**

2. In your Methods section, please provide additional information regarding the permits you obtained for the work. Please ensure you have included the full name of the authority that approved the field site access and, if no permits were required, a brief statement explaining why.

**The permission details have been added in the methods section, under the study area subsection.**

3. We note that [Figure(s) #] in your submission contain [map/satellite] images which may be copyrighted. All PLOS content is published under the Creative Commons Attribution License (CC BY 4.0), which means that the manuscript, images, and Supporting Information files will be freely available online, and any third party is permitted to access, download, copy, distribute, and use these materials in any way, even commercially, with proper attribution. For these reasons, we cannot publish previously copyrighted maps or satellite images created using proprietary data, such as Google software (Google Maps, Street View, and Earth). For more information, see our copyright guidelines: <http://journals.plos.org/plosone/s/licenses-and-copyright>.

**The required figures have been changed and updated. In Fig 1, the map for India and the part indicating Meghalaya has been taken from the** [**http://viewer.nationalmap.gov/viewer/**](http://viewer.nationalmap.gov/viewer/) **(as recommended from your end). The map for Tasek Lake has been drawn by Prof. Arup Kumar Hazarika, author of the study.**

4. Additional Editor Comments (if provided)

**We were not able to add these additional analyses due to technical issues at our end. Also, we have been badly affected by the COVID-19 pandemic, because of which we do not have the right mindset nor the appropriate resources to include these new highlights. We really apologise for this.**

**In second revision (rejection round)**

1. The preliminary round of evaluation is done to ensure that every manuscript contains a basic statistical treatment of data so that the description and the interpretarion of results would become unambiguous. For the present manuscript, one of the commonly used multivariate analyses is the Canonical Correspondence Analysis (CCA) which identifies the relation between the physico-chemical variables with species assemblages in a given ecosystem. Many workers from the region from which the present manuscript was prepared, also did the CCA analysis. For example, <https://doi.org/10.1016/j.ejar.2015.11.004> <https://doi.org/10.1007/s13201-019-1034-5>

The authors have not performed this analysis in the present work. In the absence of this or other type of multivariate analysis, it is hard to know which abiotic factors explain the variations of species abundances in different seasons. Therefore, the manuscript is not suitable for publication in this journal. However, the editor in chief gives the possibility to re-sumbit a revised version as new submission for possible consideration.

**We performed Canonical Correspondence Analysis (CCA)**
