# Supplementary material for: Ecological status of a freshwater tectonic lake of the indo-burmese province: Implications for livelihood development
Source: PLoS One. 2020 Nov 4;15(11):e0240685. doi: 10.1371/journal.pone.0240685 (PMC7641454; doi:10.1371/journal.pone.0240685)
Supplement: Attachment — Submitted filename: Response to Reviewers 23.09.20.docx [file pone.0240685.s003.docx]

**Reviewer #1:**

1. Line 74 Reference required for origin of the lake

**The reference has been added (Michael, R. G. (1975). Report on the work done during the second survey of the Tasek Lake in Caro Hills (10–13 June 1975). *School of Life Sciences, North Eastern Hill University, Shillong, India. Survey Report*, (2), 12.)**

2. Study area: details needed about depth (mean & max); inflows, outflows; climate of the area

**The data for depth and description of the area’s climate has been added. However, we do not have the precise data on inflow and outflow.**

3. Sample Collection (lines 79-87): provide information on the location and number of sampling sites in the lake; samples taken from surface or at some depth.

**Three sampling points were chosen for sampling from the lake. The samples were taken from a depth of 10-20 cm.**

4. Data are provided in the mss for every month but this is not mentioned here that sampling was done at monthly intervals.

**This has been rectified as recommended.**

5. The WQI was calculated and interpreted in context of drinking water standards. How is that relevant to plankton and fishery development? How were the diversity indices calculated?

**To standardise the study and the obtained results, drinking water standards were considered as threshold points. Further, drinking water standards are stricter than any other guidelines at present which makes it them a better set of standards for gauging overall water quality of a freshwater lake. As regards, the diversity indices, detailed techniques have been laid out in the Materials and Methods section.**

6. Any water body could be converted for fisheries but the relationship between this study and the recommendation is not clear.

**With regard to this suggestion, we would like to clarify that we have mentioned the relationship between the fishery potential of the lake and its overall ecological health. Meghalayan people depend on fishes as a source of nutritious food. Tasek lake, being surrounded by a number of villages, is a pivot spot for fishery development. Hence, the relationship between these has been clearly established in the study**

Will the fisheries protect the current fish diversity or promote a few species at the cost of most of them?

**The fisheries will protect the current fish diversity while promote species in a way congenial with the current unique ecological characteristics of the lake.**

Fig 1 has no coordinates, no mark of sampling locations and directions; hence of no value. Figure 2a, 2b have no Y axis with value and unit, and hence are wholly hypothetical and unacceptable. Fig 3a to 3d, and 4a to 4d: Y axis is not identified, what are the values? What are the letter symbols?

**These have been revised.**

In my view that tables 3-6 presenting association analysis are not required, or may be included in the supplementary material. Table 7 on fish diversity can reduced to a simple list of species.

**To present findings clearly, we have decided against removing them. However, the final decision is left to the Editor.**

The language and style of presentation need much improvement.

**This has been taken into consideration and rectified.**

**Reviewer #2:**

1. Goals are somewhat confusing; they need modification. Though field works generally lack hypothesis, I wonder if authors can make one.

**We have modified the goals in more clear terms. Since our study involves observations from extensive field study, the study doesn’t include a hypothesis.**

2. Details such as frequency procedures, sampling period, and number of replicates of each parameter are missing.

**The samples were taken in a monthly frequency for a period of 11 months (February 2019- January 2020). Samples were taken in triplicate for each parameter (3 replicates). This has been already mentioned in the Materials & Methods section.**

3. In the graphs, the mean and the standard error of how many replicates?

**These have been given for three replicates for each sample.**

4. Graphics need description of their symbols, or footnote. Taxonomic groups must be in italics. Several misspellings in phytoplankton scientific genera names.

**These have been taken note of and rectified as deemed necessary with regard to the study.**

5. Which is the monsoon period –months ?? What are the meteorologial conditions during this period.

**The months are June, July, August and September. The area receives heavy rainfall during this period, averaging on 2795.2 mm.**

6. With only slightly variation, how retreating Monsoon has positive influence on the parameters like Iron, Nitrate Phosphate and Conductivity?? In biological terms what does that mean.

**This was noticed as per the data observations from the field study. Since, our intention was not to interpret the precise water chemistry of the samples but dwell on the overall ecological health, this area is open for future research.**

7. The growth of zooplankton species (Ostracoda, Copepoda and Cladocerona) is found to be positively characterized by the high concentration level of Transparency, BOD DO, pH, water (OC) Magnesium and COD. Are these are all indirect rather than direct interactions Clarify so.

**Since, the topography and the ecological conditions of the lake are unique, the observed growth may be attributed to both direct and indirect interactions. However, we expect these interactions to be mostly indirect.**

8. Among the phytoplankton, Bacillariophyceae had the highest number of individuals in all seasons throughout the year with dominance in pre-monsoon, gradually declining towards retreating monsoon. It seems that these groups may represent the typical dynamics induced by silicates together with pH, nutrients (P, N) sources. Does this tectonic aquatic system exhibit a particular mineral physico-chemical environment??

**As far as previous literature is concerned, the system does exhibit a physico-chemical environment. However, this environment is being continuously altered due to surrounding ecological alterations.**

9. How is that the diversity indices and the environmental conditions are associated with fisheries potential. Explain or delete it.

**The diversity indices indicate the planktonic diversity of the lake. Changes in their diversity can lead to adverse changes in the overall environmental health of the lake. This could ultimately destroy the fishery potential of the lake. For instance, overly grown phytoplankton can drastically reduce the dissolved oxygen content of the water which will ultimately damage the aquatic life within. Hence, diversity indices indicate such changes which may later affect the ecological health and the fishery potential of a water body.**

10. Unnecessary over citation and outdated literature. As far as possible cite only indexed articles. Popular articles, newspaper statements must be avoided and they are rigorously evaluated by peer review processes.

**The manuscript has been accordingly revised.**

**Reviewer #3:**

1. Line 134 – how much litre of water filtered through net?

**20 litres of water were filtered through the net for plankton collection.**
